# Therapeutic Potential of Gum Arabic (*Acacia senegal*) in Chronic Kidney Disease Management: A Narrative Review

**DOI:** 10.3390/jcm13195778

**Published:** 2024-09-27

**Authors:** Sami Alobaidi

**Affiliations:** Department of Internal Medicine, University of Jeddah, Jeddah 21493, Saudi Arabia; salobaidi@uj.edu.sa

**Keywords:** chronic kidney disease, gum arabic, *Acacia senegal*, renal health, traditional medicine

## Abstract

Chronic kidney disease (CKD) poses significant health challenges globally, particularly in regions like the Middle East. This review evaluates the potential efficacy and safety of Gum Arabic (*Acacia senegal*), a traditional remedy, in managing CKD. A comprehensive literature review was conducted using databases including PubMed and Scopus, focusing on the biochemical, physiological, and therapeutic impacts of Gum Arabic on renal health. Gum Arabic has demonstrated antioxidative and anti-inflammatory properties that may benefit renal function, as shown in animal studies. Clinical trials suggest improvements in renal biomarkers, though these are limited by scope and methodology. While promising, the clinical application of Gum Arabic requires cautious interpretation due to gaps in understanding its mechanisms of action. Gum Arabic shows potential as an adjunct treatment for CKD, reflecting both traditional use and preliminary scientific evidence. Future research should focus on its long-term efficacy, safety, and underlying biochemical pathways to better guide its therapeutic use.

## 1. Introduction

Chronic kidney disease (CKD) is a significant public health challenge globally, with its rising incidence particularly linked to risk factors such as diabetes and hypertension, especially in regions like the Middle East. CKD can result from various underlying causes, including inherited disorders and systemic diseases, contributing to the complexity of its management. Despite these diverse causes, the primary concern remains the progressive decline in kidney function and the urgent need for effective treatment strategies [1].

In response to the growing burden of CKD, there is increasing interest in integrating natural therapies with conventional treatments. Among these, Gum Arabic (GA), derived from the sap of the *Acacia senegal* tree, has attracted attention for its prebiotic, anti-inflammatory, and antioxidant properties, which may offer therapeutic benefits in slowing CKD progression [2,3].

This review aims to evaluate the integration of traditional remedies like GA into modern medical practices, specifically focusing on its efficacy and safety in CKD management. By examining evidence from both animal and human studies, we provide a comprehensive analysis of GA’s potential benefits, particularly in regions where traditional remedies are culturally significant. We also highlight promising areas of GA’s application and identify gaps in current research that warrant further investigation.

In conducting a comprehensive literature review, we begin by examining GA’s historical use and fundamental properties to better understand its role in CKD management. This review offers a nuanced assessment of GA’s therapeutic potential and its relevance to modern treatment strategies, while outlining areas for future research.

## 2. Methods

This review critically examines the current literature on the therapeutic effects of GA on CKD, focusing on its biochemical, physiological, and therapeutic impacts. We conducted a systematic literature search using databases such as PubMed, Scopus, and Web of Science. The search strategy included keywords like “Gum Arabic”, “*Acacia senegal*”, “kidney disease”, and “renal function”. We selected peer-reviewed articles published in English, emphasizing experimental and clinical trials, as well as review articles and meta-analyses that enrich the understanding of GA’s role in CKD management.

The data extraction and synthesis were performed by the author, aiming to integrate significant findings from both animal and human studies to provide a comprehensive overview of the evidence. This approach ensured a structured presentation of the potential benefits and limitations of GA in CKD, adhering to the narrative review guidelines.

It is important to note that the exclusion of non-English studies may introduce publication bias, limiting the review’s scope to predominantly English-language research. This restriction necessitates cautious interpretation of the findings, particularly in understanding the global context of the research.

## 3. Gum Arabic: Overview and Properties

### 3.1. Historical Use and Significance

Gum Arabic, also known as acacia gum, is harvested from the sap of the *Acacia senegal* and *Acacia seyal* trees. It has been used for centuries in traditional medicine, as well as in various industries including food, pharmaceuticals, and cosmetics. In the Middle East and Africa, it has traditionally been used to manage gastrointestinal issues, obesity, and diabetes. More recently, its use has been explored in the management of CKD, where it is valued for its therapeutic properties [4,5].

### 3.2. Chemical Composition

GA is a complex mixture of polysaccharides and glycoproteins, which confer its high solubility in water and minimal viscosity. The main components are arabinogalactan proteins, known for their range of biological activities. These components consist of a branched-chain, complex polysaccharide structure predominantly made up of 1,3-linked β-D-galactopyranosyl units with side chains that influence their functional properties, including potential anti-inflammatory effects beneficial in renal protection [6]. These structures contribute to the prebiotic properties of GA by fostering gut microbiota that can significantly affect systemic health and are specifically beneficial in managing CKD [7]. The modulation of various metabolic pathways by these polysaccharides and glycoproteins is vital for overall health and particularly advantageous for CKD management.

### 3.3. Safety Profile

GA is widely recognized as safe for human consumption and has been classified as “Generally Recognized As Safe” (GRAS) by the FDA [8]. Animal studies have consistently supported its safety, showing no significant adverse effects even at high dietary levels [8,9,10]. Human trials have further validated its safety, demonstrating that GA is well tolerated with no significant adverse effects, while also providing health benefits such as reducing inflammatory markers and improving hepatic and renal profiles [11,12,13].

### 3.4. Biological Activities

The efficacy of GA in various health applications is due to its diverse biological activities. Notably, GA fosters the growth of beneficial gut bacteria such as bifidobacteria and lactobacilli, improving gut health and potentially impacting systemic health conditions including CKD. The influence of GA on the intestinal microbiome has been demonstrated in experimental studies with CKD models in rats, where it significantly promoted the growth of these beneficial bacteria, enhancing gut health and offering a potential therapeutic avenue for CKD management [14]. Additionally, a randomized, double-blinded, double-controlled trial in healthy human volunteers confirmed the prebiotic efficacy of GA, showing significant increases in bifidobacteria and lactobacilli in a dose-dependent manner, with the optimal dose being 10 g daily [15]. GA also modulates cytokine production and combats oxidative stress through its antioxidant properties, both of which are vital in managing CKD and its complications. These properties make GA a candidate for further research into its potential benefits across a spectrum of chronic conditions, particularly those involving inflammation and oxidative stress. Understanding these properties provides a foundation for exploring the biochemical mechanisms through which GA exerts its effects on renal health [14,16].

These properties make GA a candidate for further research into its potential benefits across a spectrum of chronic conditions, particularly those involving inflammation and oxidative stress. Understanding these properties provides a foundation for exploring the biochemical mechanisms through which GA exerts its effects on renal health.

## 4. Mechanisms of Action of Gum Arabic in Renal Health

GA has garnered significant attention in scientific research due to its potential therapeutic effects, particularly concerning renal health. This section outlines the various biochemical pathways and physiological mechanisms through which GA may exert its beneficial effects, emphasizing both animal model insights and clinical observations from human studies.

### 4.1. Biochemical Pathways and Renal Protection

**Figure 1:**
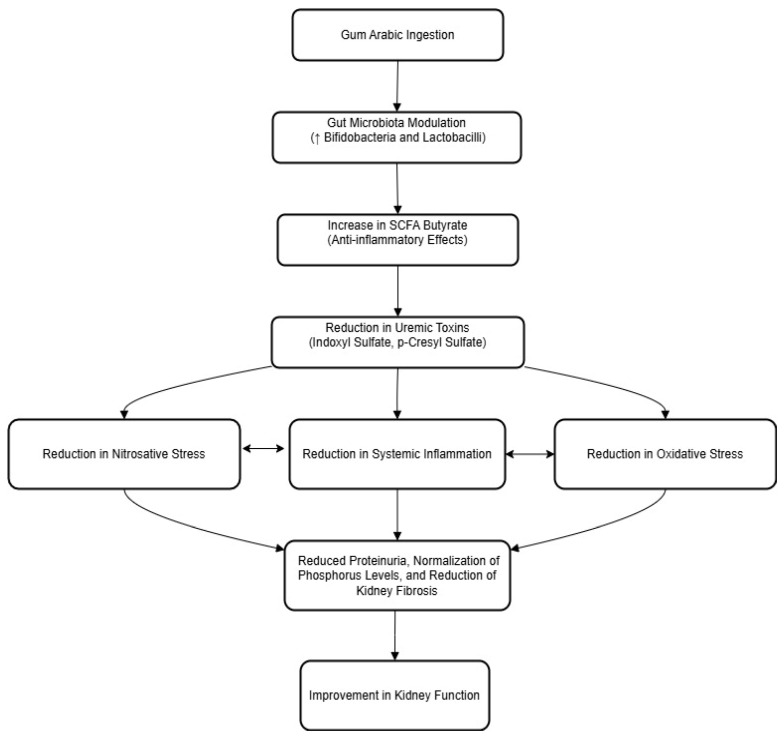
Biochemical pathways of Gum Arabic’s impact on gut microbiota and renal protection. Up arrow, increase.

GA, recognized for its significant prebiotic properties, promotes the growth of beneficial gut bacteria such as bifidobacteria and lactobacilli. This enhancement in gut microbiota composition is pivotal for improving overall health and specifically managing CKD. The study by Calame et al. (2008) demonstrates that GA establishes its prebiotic functionality in a dose-dependent manner, significantly increasing beneficial bacteria after consumption, which can influence systemic health including renal function [15]. Furthermore, Lakshmanan et al. (2021) explored the prebiotic effects of GA on the intestinal microbiome composition in a rat model of CKD, finding that GA not only adjusts the microbial balance but also restores depleted butyrate levels, a critical short-chain fatty acid with anti-inflammatory properties. These biochemical interactions suggest a therapeutic pathway whereby GA can mitigate uremic toxins and enhance renal health, emphasizing its potential as an integrative approach to CKD management [14]. The detailed biochemical pathways showing how GA modulates the gut microbiome and influences renal protection are summarized in Figure 1.

### 4.2. Anti-Inflammatory and Antioxidant Effects

The anti-inflammatory and antioxidant properties of GA are essential in its potential to manage CKD. Chronic inflammation exacerbates kidney damage, and GA has been shown to reduce systemic inflammatory cytokines effectively. In an adenine-induced CKD rat model, B.H. Ali et al. (2013) and Al Za’abi et al. (2015) demonstrated that GA reduces markers of inflammation and oxidative stress, enhances antioxidant capacity, and improves renal function [16,17]. The antioxidative mechanism of GA involves its impact on the Nrf2 signaling pathway, which increases antioxidant enzymes such as superoxide dismutase (SOD), catalase (CAT), and glutathione peroxidase (GPx) in renal tissues.

In addition, Al-Majed et al. (2003) reported that GA reduces lipid peroxidation in cisplatin-induced nephrotoxicity, suggesting that GA can scavenge free radicals to protect renal tissues from oxidative damage [18]. GA has also been shown to inhibit the activation of the NF-κB pathway, thereby reducing the production of pro-inflammatory cytokines, such as TNF-α and IL-6. In clinical trials involving rheumatoid arthritis patients, GA consumption led to significant reductions in TNF-α levels and erythrocyte sedimentation rate (ESR), along with an improvement in disease activity [19].

Recent studies further highlight GA’s antioxidative potential in models of high-fat-diet-induced oxidative stress, where GA increased antioxidant enzyme activities and reduced oxidative stress markers in mice [20]. In another study, GA ameliorated renal damage in an adenine-induced chronic renal failure model by modulating the Keap1-Nrf2 signaling pathway, thus improving the body’s antioxidative defenses [21]. Additionally, GA’s influence on immune response includes enhancing cathelicidin expression in monocyte-derived macrophages, which boosts the innate immune response and reduces inflammation [22]. These findings confirm GA’s therapeutic potential in managing CKD through its antioxidative and anti-inflammatory effects.

### 4.3. Improvement in Renal Hemodynamics

Improvements in renal blood flow and vascular function attributed to have been well documented predominantly in animal models. These effects are significant as they help maintain kidney perfusion and function under compromised conditions, such as those seen in CKD and diabetic nephropathy. Al Suleimani et al. (2014) demonstrated that GA mitigates impairment in renal vascular responses to vasoactive stimuli in a rat model of adenine-induced CKD, potentially through the modulation of inflammatory pathways and oxidative stress [23]. Furthermore, Mohammed et al. (2020) evaluated GA’s impact on vascular mediators like TGF-β1, endothelin-1, and angiotensin II in diabetic nephropathy, showing that GA significantly reduced these mediators, which are known to contribute to vascular dysfunction and renal damage [24]. Collectively, these findings suggest that GA not only supports renal hemodynamics but also has therapeutic potential in managing the vascular complications associated with renal diseases.

### 4.4. Clinical Relevance and Integration into CKD Management

The integration of these mechanisms into clinical practice could offer significant benefits for CKD management. Regular supplementation with GA has been demonstrated to potentially stabilize kidney function and improve overall health outcomes for patients with CKD. For instance, Khalid et al. (2021) observed that GA supplementation was associated with a significant initial increase in eGFR, followed by stabilization, suggesting its role in slowing the progression of renal disease in early-stage CKD patients [25]. Additionally, Nour Elkhair Ali et al. (2020) found that GA significantly increased total antioxidant capacity and decreased malondialdehyde (MDA) and C-reactive protein (CRP) levels, indicating potent antioxidative and anti-inflammatory properties [26]. Studies on diabetic rats have demonstrated that Gum Arabic, either alone or combined with Folium mori, significantly improved glycemic control, reduced oxidative stress, and enhanced liver and kidney function, reinforcing its potential role in ameliorating complications related to CKD [27,28]. Furthermore, a randomized placebo-controlled trial in T2DM patients showed that GA significantly improved glycemic control, lipid profile, and BMI, further supporting its potential benefits in managing metabolic complications in CKD patients [29]. Additionally, a study showed that GA treatment decreased SGLT1 protein abundance, reduced intestinal glucose absorption, and mitigated glucose-induced obesity, indicating its potential to manage metabolic complications in CKD patients [30]. These clinical trials highlight GA’s potential to improve biochemical markers of kidney function and reduce the progression of renal disease, supporting its integration into therapeutic strategies for CKD management.

## 5. Results

### 5.1. Efficacy of Gum Arabic in Renal Function Improvement: Insights from Animal Studies

The therapeutic potential of GA in mitigating kidney disease has been robustly demonstrated across various animal models, as summarized in Table 1. A significant body of research shows marked improvements in renal function markers such as serum creatinine, urea, and enhanced creatinine clearance. Studies consistently report the antioxidative and anti-inflammatory properties of GA, emphasizing its role in reducing the progression factors of kidney disease. The extensive contributions from research groups, particularly those led by B. H. Ali [6], refs. [16,17,21,31,32,33,34,35,36,37,38,39,40,41,42] have been pivotal in illustrating these effects, although the need for broader research involvement remains to diversify and validate these findings across different experimental setups and conditions.

### 5.2. Impact of Gum Arabic on Renal Health: A Synthesis of Human Clinical Trials

Human clinical trials have further explored the potential benefits of GA, particularly in its ability to manage blood pressure and improve biochemical markers related to kidney function, as detailed in Table 2. Significant findings include the modulation of blood pressure, as reported by Glover et al. (2009), who observed a notable reduction in systolic blood pressure in both healthy individuals and patients with diabetic nephropathy, without adversely affecting renal function [43].

Moreover, studies like those conducted by Kaddam et al. (2019) and Kamal et al. (2021) demonstrated GA’s potential in improving liver and renal profiles and reducing inflammatory markers in patients with various systemic conditions, suggesting its broad applicability beyond CKD management [11,44].

### 5.3. Limitations of Human Studies

Despite promising results, the human studies reviewed exhibit significant limitations that must be acknowledged. These include small sample sizes, heterogeneous populations, variable dosages, and relatively short study durations, all of which complicate the interpretation of the findings. Furthermore, the lack of control groups in some studies and insufficient monitoring for compliance and adverse effects highlight the need for more rigorously designed trials.

### 5.4. Concluding Observations

While the results from animal studies provide a strong basis for GA’s efficacy in renal protection, human clinical trials, though supportive, underscore the need for further research. The existing data suggest potential benefits in renal health, but the evidence is not yet robust enough to recommend GA for broad clinical use. Continued research efforts are essential to provide a more definitive understanding of its efficacy and safety in CKD management.

**Table 1:** Summary of animal studies investigating the effects of Gum Arabic on renal function.

#	Study Reference	Animal Model	Dosage of GA and Duration	Major Findings
1	Al-Majed et al., 2002 [45]	Rats, gentamicin-induced nephrotoxicity	7.5 g/100 mL in drinking water, 8 days	GA reduced serum creatinine and urea levels, improved creatinine clearance, decreased MDA levels, and improved histopathological changes.
2	Al-Majed et al., 2003 [18]	Rats, cisplatin-induced	7.5 g/kg/day, 5 days	GA reduced cisplatin-induced lipid peroxidation but did not protect against kidney damage in terms of glutathione depletion or platinum accumulation.
3	B. H. Ali et al., 2003 [38]	Rats, gentamicin-induced	10% *w*/*v*, 2 mL/kg/day, 10 days	GA modestly ameliorated gentamicin-induced increases in creatinine and urea and the histological damage in the kidneys.
4	B. H. Ali et al., 2004 [31]	Rats, two-stage surgical nephrectomy	3 or 6 g/100 mL/day, 5 weeks	Slight and insignificant reductions in plasma urea and creatinine concentrations; no significant impact on body weight loss in CRF rats.
5	Nasir et al., 2008 [46]	Mice, healthy	10% in drinking water, 3 weeks	GA increased creatinine clearance and significantly altered electrolyte excretion, leading to improved overall water and electrolyte balance in healthy mice.
6	B. H. Ali et al., 2010 [33]	Rats, adenine-induced	6% or 12% *w*/*v* in drinking water, 4 weeks	GA improved renal function, reduced urea and creatinine levels, and ameliorated histopathological changes in kidneys.
7	B. H. Ali et al., 2011 [36]	Rats, adenine-induced	10% *w*/*v* in drinking water, 4 weeks	GA mitigated motor and behavioral changes induced by CKD, and improved renal function parameters.
8	B. H. Ali et al., 2011 [41]	Rats, adenine-induced	10% *w*/*v* in drinking water, 4 weeks	GA reduced plasma urea and creatinine levels, improved creatinine clearance, and mitigated increases in blood pressure.
9	Nasir et al., 2012 [47]	Mice, diabetic Akita	10% in drinking water, 3 weeks	GA treatment decreases blood pressure and proteinuria in diabetic mice, and may prove beneficial in diabetic nephropathy.
10	Mahmoud et al., 2012 [48]	Rats, adenine-induced CRF and ischemia–reperfusion injury	200 mg/kg orally, 8 weeks	GA reduced serum creatinine, urea, BUN, LDH, and MDA levels, and increased renal SOD activity.
11	Gado and Aldahmash 2013 [49]	Rats, mercuric chloride-induced	7.5 g/kg body weight/day, 1 week	GA prevented Hg-induced increases in creatinine and BUN, improved oxidative stress markers, and histopathological changes.
12	B. H. Ali et al., 2013a [50]	Rats, adenine-induced	15% *w*/*v* in drinking water, 4 weeks	GA ameliorated signs of CKD similarly across three different brands tested, reducing oxidative stress and inflammation.
13	B. H. Ali et al., 2013b [16]	Rats, adenine-induced	15% *w*/*v* in drinking water, 4 weeks	GA significantly reduced inflammation, oxidative stress, and DNA damage, and improved renal histopathology.
14	B. H. Ali et al., 2013c [21]	Mice and rats, adenine-induced CRF	15% *w*/*v* in drinking water, 4 weeks	GA reduced plasma urea and creatinine levels, urinary protein, and apoptosis. It improved renal SOD activity, GSH concentration, and histopathological changes.
15	Suleimani et al., 2014 [23]	Rats, adenine-induced	15% *w*/*v* in drinking water, 5 weeks	GA improved RBF, mitigated the impaired vascular responses, and reduced biochemical markers of CKD.
16	B. H. Ali et al., 2014 [51]	Rats, adenine-induced	15% *w*/*v* in drinking water, 4 weeks	GA mitigated myocardial and renal damage in CKD, showing decreased myocardial hypertrophy and interstitial fibrosis.
17	B. H. Ali et al., 2014 [32]	Rats, adenine-induced	15% *w*/*v* in drinking water, 5 weeks	GA and swimming exercise combined significantly improved CKD indices, including reductions in serum biomarkers and enhancements in renal histopathology.
18	B. H. Ali et al., 2014 [40]	Rats, adenine-induced	15% *w*/*v* in drinking water, 4 weeks	Ameliorated anemia, reduced serum creatinine and urea levels, improved creatinine clearance and renal histopathology.
19	Al Za’abi et al., 2015 [17]	Rats, adenine-induced	15% *w*/*v* ( weight/volume) in drinking water, 4 weeks	GA significantly mitigated all indices of CKD, including oxidative and inflammatory markers, and renal damage.
20	B. H. Ali et al., 2015 [34]	Rats, adenine-induced	15% *w*/*v* in drinking water, 4 weeks	GA mitigated genetic damage in renal tissues and reduced the level of nucleic acid oxidation markers in urine.
21	Al Suleimani et al., 2016 [42]	Rats, adenine-induced CKD and DP exposure	15% *w*/*v* in drinking water, 4 weeks	GA reduced plasma urea and creatinine levels, improved creatinine clearance, and mitigated vascular impairment and renal damage caused by DP and CKD.
22	B. H. Ali et al., 2018 [37]	Rats, potassium bromate-induced	15% *w*/*v* in drinking water, 4 weeks	GA significantly reduced renal damage, inflammation, and oxidative stress induced by potassium bromate.
23	Hammad et al., 2019 [52]	Rats, reversible unilateral ureteric obstruction	15 g/kg/day in drinking water, 13 days	GA attenuated UUO-induced oxidative stress markers, gene expression of TNF-α, TGF-β1, p53, and tubular dilatation. It improved renal tubular function but did not significantly affect RBF or GFR.
24	Shafeek et al., 2019 [53]	Rats, diclofenac-induced nephrotoxicity	1, 2, and 3 g/kg/day in drinking water, 12 weeks	GA reduced serum creatinine, urea, uric acid, MDA levels, IL-1β, TNF-α, MCP-1, and caspase-3. It increased catalase, GSH, IL-10, and CR-1.
25	Albeladi 2019 [54]	Rats, glycerol-induced nephrotoxicity	3 mL/kg in drinking water, 4 weeks	GA reduced serum creatinine levels, improved histopathological changes, reduced inflammation and protein accumulation in the kidneys.
26	B. H. Ali et al., 2020 [35]	Mice, adenine-induced	15% *w*/*v* in drinking water, 4 weeks	GA reduced inflammation, and duodenal oxidative and nitrosative stress in CKD mice.
27	Mohammed et al., 2020 [24]	Rats, streptozotocin-induced diabetic nephropathy	10% *w*/*v* in drinking water, 12 weeks	GA significantly decreased serum glucose, creatinine, TGF-β1, angiotensin II, and endothelin-1 levels in diabetic rats. Insulin combined with GA had a synergistic effect in reducing these parameters.
28	Al-Asmakh et al., 2020 [55]	Rats, adenine-induced	15% *w*/*v* in drinking water, 4 weeks	GA improved gut microbiome diversity and SCFA levels, and mitigated renal dysfunction indicators.
29	Lakshmanan et al., 2020 [14]	Rats, adenine-induced	15% *w*/*v* in drinking water, 4 weeks	GA influenced the microbiome composition beneficially, improving renal function and gut wall integrity.
30	Al Za’abi et al., 2020 [39]	Rats, streptozotocin-induced diabetic nephropathy with adenine-induced CKD	15% *w*/*v* in drinking water, 4 weeks	GA significantly improved renal function and reduced inflammation and oxidative and nitrosative stress in diabetic nephropathy models, leading to decreased kidney tissue damage
31	El-Garawani et al., 2021 [56]	Rats, Ioxitalamate-induced nephrotoxicity	10% *w*/*v* in drinking water, 14 days	GA reduced serum urea and creatinine levels, decreased MDA and NO levels, increased CAT and GSH activities, and ameliorated DNA damage and chromosomal aberrations.
32	Refaie et al., 2021 [57]	Rats, butralin-induced	4.3 g/kg b.wt, 30 days	GA ameliorated butralin-induced increases in serum creatinine and BUN, reduced DNA damage, and normalized gene expression related to kidney function.
33	Bashir et al., 2022 [58]	Rats, streptozotocin-induced	20 g/kg body weight, 12 weeks	Significant improvement in serum creatinine and creatinine clearance. Histological improvements in kidney tissue.
34	Shanab et al., 2023 [59]	Rats, aflatoxin B1 exposure	7.5 mg/kg, 4 weeks	GA protected against aflatoxin B1-induced renal damage, inflammation, and apoptosis.
35	Al-Ghamdi et al., 2023 [60]	Rats, furosemide-induced nephrotoxicity (newborn)	15% *w*/*v* in drinking water, from conception to 2 weeks postpartum	GA reduced serum creatinine and urea levels, decreased MDA levels, increased GSH levels, and improved histopathological changes.

**Table 2:** Summary of human clinical trials evaluating the effects of Gum Arabic on renal function.

#	Study Reference	Population	Dosage of GA and Duration	Major Findings
1	Bliss et al., 1996 [61]	16 chronic renal failure patients consuming a low-protein diet	50 g/day for 4 weeks	GA significantly increased fecal nitrogen excretion and decreased serum urea nitrogen concentration compared to baseline and placebo.
2	Matsumoto et al., 2006 [7]	10 healthy volunteers	25 g/day for 10 weeks	Dietary supplementation with GA increased serum butyrate levels and suppressed TGF-b1 generation and its signaling pathways.
3	Glover et al., 2009 [43]	10 healthy individuals, 37 diabetic nephropathy patients	25 g daily for 8–12 weeks	Significant reduction in systolic blood pressure in healthy and diabetic patients; no effect on renal function in diabetic nephropathy patients
4	Adil Ahmed Ali et al., 2008 [62]	36 chronic renal failure patients under regular hemodialysis	50 g/day for 3 months	Significant reduction in serum urea and creatinine levels; changes in uric acid, calcium, and phosphorus levels after treatment with GA.
5	Elamin et al., 2017 [12]	30 CKD patients, stage 3B/4	10, 20, or 40 g daily for 4 weeks	GA significantly reduced CRP levels; no effect on urea, creatinine, or indoxyl sulfate levels. Minimal impact on serum sodium levels.
6	Kaddam et al., 2019 [11]	47 patients with sickle cell anemia	30 g/day for 12 weeks	GA decreased direct bilirubin and urea levels; temporary decrease in ALT levels.
7	NE Ali et al., 2020 [26]	40 end-stage renal failure patients on regular hemodialysis	30 g/day for 12 weeks	GA significantly increased TAC and decreased MDA and CRP levels, indicating potent antioxidative and anti-inflammatory properties.
8	Kamal et al., 2021 [44]	40 rheumatoid arthritis patients	30 g/day for 12 weeks	GA significantly decreased liver enzymes and urea levels, increased serum albumin, and had minor impacts on serum globulin levels. No significant change in alkaline phosphatase.
9	Khalid et al., 2021 [25]	68 patients with stage 2 or 3 chronic kidney disease	25 g/day for 12 months	GA supplementation was associated with a significant initial increase in eGFR followed by stabilization, leading to a slower progression of renal disease.

## 6. Clinical Implications and Future Research Directions

### 6.1. Clinical Implications of Gum Arabic in CKD Management

GA has shown potential in renal health management through both animal and human studies, demonstrating antioxidative and anti-inflammatory properties. These studies suggest that GA could potentially enhance renal function markers, such as serum creatinine and urea levels, as observed in animal models [16,24,33,38,39,46,58]. However, the translation of these effects into clinical practice remains uncertain due to the limited scope and scale of human studies, as outlined in Table 2. While some results appear promising, they reflect the complexity of directly applying experimental outcomes to patient care.

Given the current evidence, healthcare professionals should be prepared to discuss GA with patients, especially when asked about its use. It is important to convey that although preliminary results are encouraging, GA should not be considered a standalone treatment for CKD. Instead, it might be discussed as part of an exploratory approach within a broader, integrative treatment strategy, particularly for patients in the early stages of CKD seeking to slow disease progression.

Healthcare providers should emphasize that the existing data do not yet support robust clinical recommendations for GA, underscoring the need for further research to establish its efficacy and safety in CKD management more definitively.

### 6.2. Addressing Mechanistic Limitations

While GA shows promise in managing CKD, a significant barrier to its clinical application is the incomplete understanding of its underlying mechanisms. This gap complicates its full integration into clinical practice, as it obscures the ability to foresee long-term effects, potential interactions with standard CKD medications, and optimal dosing strategies. Particularly, the pharmacokinetics of GA in CKD patients and how it is absorbed, processed, and cleared from the body remain poorly understood. Thus, clinical adoption should proceed with caution, with therapeutic decisions grounded in a holistic assessment of individual patient profiles and rigorous monitoring of clinical outcomes. Future research should prioritize filling these mechanistic voids, employing an interdisciplinary approach to better elucidate how GA exerts its renal protective effects. This understanding is crucial for safely harnessing GA’s full potential in CKD management, ensuring that treatment protocols are both effective and devoid of unforeseen complications.

### 6.3. Future Research Directions

The promising potential of GA in renal health underscores the need for robust, targeted research to validate its efficacy and safety. Essential future investigations should include the following:

Longitudinal and Large-Scale Clinical Trials: There is a critical need for well-designed, long-term studies involving diverse populations to assess the comprehensive effects of GA. These studies should include randomized control groups, blind assessments, and the tracking of specific clinical outcomes to establish standardized treatment protocols effectively. In particular, the dosage of GA, the duration of supplementation, and the inclusion of standardized biomarkers such as CRP, urea, creatinine, and GFR should be consistent across trials to ensure the reliability and comparability of results.

Mechanistic Studies: Further research is imperative to elucidate the specific biochemical and molecular pathways by which GA confers renal protection. Targeted studies should explore its anti-inflammatory effects, potential to reduce fibrosis, and interactions with renal function markers.

Quality of Life and Patient Satisfaction Studies: It is also vital to assess the impact of GA on the quality of life and overall patient satisfaction. Such studies will help determine the real-world applicability of GA in routine CKD management, assessing both subjective and objective health benefits.

Comparative Studies: Comparative research should be conducted to evaluate GA against other prebiotics and renal-protective agents. These studies will provide critical insights into its relative efficacy and suitability for various patient groups, potentially identifying unique patient cohorts who might benefit most from its use. In addition, studies investigating combination therapies, where GA is used alongside established CKD treatments such as phosphate binders, RAS inhibitors, SGLT2Is, GLP1RAs, or erythropoiesis-stimulating agents, could explore potential synergistic effects. Such research could further enhance our understanding of how GA may optimize treatment outcomes in CKD management.

Interdisciplinary Research Approaches: Collaborative research integrating nephrology, pharmacology, nutrition science, immunology, and molecular biology is essential for advancing GA’s role in CKD management. Nephrology will focus on renal function effects, while pharmacology explores interactions with CKD medications. Nutrition science can assess GA’s prebiotic influence, and immunology will clarify its anti-inflammatory mechanisms.

Molecular biology is crucial for investigating pathways like Nrf2 and NF-κB. Future studies should prioritize randomized controlled trials (RCTs), supported by omics approaches and pharmacokinetic studies to understand GA’s molecular impact and drug interactions. Gut microbiome analysis can further explore the link between GA and kidney health.

### 6.4. Communicating Uncertainties

It is crucial to maintain a balanced perspective when discussing the potential of GA in the management of chronic kidney disease, both with patients and within the scientific community. Highlighting the preliminary nature of current research findings is essential to set realistic expectations and foster an evidence-based approach to treatment. The existing research on GA shows promise but also reveals significant gaps, particularly in our understanding of its mechanisms and long-term effects.

Given the insufficiency of current data, healthcare providers should remain cautious and not yet integrate GA into clinical practice as a recommended treatment. Instead, they should be well informed about the existing studies to discuss this topic knowledgeably when approached by patients who may have heard about or are interested in using GA. Providers should emphasize that while the initial results are encouraging, they are preliminary, and GA should not replace established CKD treatments.

Ongoing research is critically needed to provide a more definitive assessment of the efficacy and safety of GA. Until more substantial evidence is available, it should not be considered a standard therapeutic option. The future clinical guidelines and recommendations will be shaped by rigorous, well-conducted studies that are yet to be completed.

## 7. Conclusions

GA has demonstrated significant promise in managing CKD through its antioxidative and anti-inflammatory effects, which may beneficially impact renal function. However, despite these encouraging results, the current evidence base is insufficient to formally endorse GA as a standard therapeutic intervention for CKD patients. Significant research gaps remain, particularly regarding the long-term safety and interactions of GA with standard CKD medications.

Continued research is crucial to substantiate these preliminary findings. Future studies should focus on long-term clinical trials involving diverse populations, detailed mechanistic studies, and evaluations of pharmacological interactions to clarify GA’s optimal use and potential integration into broader CKD management strategies. Such research should adopt an interdisciplinary approach, integrating expertise from multiple healthcare fields to ensure a holistic understanding of the benefits and risks associated with GA.

As the scientific community advances our knowledge through rigorous investigation, it will become possible to make more definitive recommendations regarding the incorporation of GA into CKD treatment regimens. Healthcare providers should remain cautious, prioritizing established care practices and considering new treatments only when a robust body of evidence supports their efficacy and safety. The future of CKD management may well incorporate natural remedies like GA, provided that their use is grounded in solid scientific validation and aligned with patient-centered care principles.

## Data Availability

Not applicable as this is a review article.
